# EEG Source Network for the Diagnosis of Schizophrenia and the Identification of Subtypes Based on Symptom Severity—A Machine Learning Approach

**DOI:** 10.3390/jcm9123934

**Published:** 2020-12-04

**Authors:** Jeong-Youn Kim, Hyun Seo Lee, Seung-Hwan Lee

**Affiliations:** 1 Center for Bionics, Korea Institute of Science and Technology (KIST), Seoul 02792, Korea; jeongyounk@gmail.com; 2 Clinical Emotion and Cognition Research Laboratory, Inje University, Goyang 10380, Korea; jslvfe77@gmail.com; 3 Department of Psychiatry, Inje University, Ilsan-Paik Hospital, Goyang 10380, Korea

**Keywords:** schizophrenia, subtypes, PANSS (positive and negative syndrome scale), EEG (electroencephalography), machine learning

## Abstract

A precise diagnosis and a comprehensive assessment of symptom severity are important clinical issues in patients with schizophrenia (SZ). We investigated whether electroencephalography (EEG) features obtained from EEG source network analyses could be effectively applied to classify the SZ subtypes based on symptom severity. Sixty-four electrode EEG signals were recorded from 119 patients with SZ (53 males and 66 females) and 119 normal controls (NC, 51 males and 68 females) during resting-state with closed eyes. Brain network features (global and local clustering coefficient and global path length) were calculated from EEG source activities. According to positive, negative, and cognitive/disorganization symptoms, the SZ patients were divided into two groups (high and low) by positive and negative syndrome scale (PANSS). To select features for classification, we used the sequential forward selection (SFS) method. The classification accuracy was evaluated using 10 by 10-fold cross-validation with the linear discriminant analysis (LDA) classifier. The best classification accuracy was 80.66% for estimating SZ patients from the NC group. The best classification accuracy between low and high groups in positive, negative, and cognitive/disorganization symptoms were 88.10%, 75.25%, and 77.78%, respectively. The selected features well-represented the pathological brain regions of SZ. Our study suggested that resting-state EEG network features could successfully classify between SZ patients and the NC, and between low and high SZ groups in positive, negative, and cognitive/disorganization symptoms.

## 1. Introduction

Schizophrenia (SZ) has been primarily diagnosed based on diagnostic criteria from the Diagnostic and Statistical Manual of Mental Disorders (DSM-5) by asking patients a series of questions designed to elicit information, such as the duration of illness and presence of clinical symptoms [1]. Various diagnostic tools can aid psychiatrists and clinical psychologists in diagnosing SZ, but traditional clinical diagnoses might sometimes be inaccurate because SZ patients may intentionally obscure their symptoms, and even specialists often have difficulty distinguishing SZ from other psychoses due to similarities of symptoms [2,3,4]. Thus, several researchers have attempted to improve the overall accuracy of diagnosis by developing objective quantitative biomarkers using neuroimaging technologies. Electroencephalography (EEG) is considered the most effective neuroimaging modality among the various neuroimaging methods due to its high time resolution and low cost. A number of studies reported disruption of cortical information processing in SZ, based on distorted functional connectivity networks [5,6] and decreased source activity [7,8,9,10].

SZ is perceived as a complex illness portrayed by heterogeneous symptoms. Over recent decades, it has been perceived that schizophrenic symptoms in fact go beyond the dichotomous divisions of positive versus negative domains [11]. The Positive and Negative Syndrome Scale (PANSS) is one of the most broadly utilized measures to assess the severity of positive symptoms and negative symptoms in SZ research [12]. Other clinical tools, such as the Brief Negative Symptom Scale (BNSS) [13,14] and Schedule for the Deficit Syndrome (SDS) [15], can be used to assess negative symptoms. Cognitive deficits have been emphasized as one of the important features of SZ [16]. The Consortium to Establish a Registry for Alzheimer’s Disease (CERAD) [17] and Mini-Mental State Examination (MMSE) [18] could be used to assess such cognitive symptoms. This cognitive impairment is a broader concept than specific clinical disorganization symptoms. The clinical/disorganization domain is also one of the fundamental aspects of SZ [19]. The cognitive disorganization could be assessed by the PANSS with a five-factor model, which was created based on several factor analysis studies [20,21]. The cognitive/disorganization factor delineates a cognitive dimension, as evidenced by the expression of the constituent symptoms, including conceptual disorganization, difficulty in abstract thinking, poor attention, disorientation, and preoccupation [21].

Thus far, the psychiatric symptom severity of SZ has generally been assessed using psychological measures, such as the PANSS [22], based on interview-based assessments conducted by trained interviewers. Although these assessments have been reported to provide high inter-rater reliability or test/retest reliability [12,23], the results might be highly affected by original diagnosis [24] or biased views on the part of the psychiatrists [4]. In addition, the negative symptoms of SZ are more difficult to evaluate [2,4], because negative symptoms may have no clear signs or common behaviors that overlap with other mental diseases [25], and the negative symptoms are divided into two sub-domains: expressive deficits and social amotivation [26]. The assessment of symptom severity in SZ is critical in establishing successful treatment strategies or evaluating the effectiveness of treatments. However, only few quantitative diagnosis tools exist to evaluate the symptom severity of individual patients with SZ [27].

Previous studies reported that, compared to normal control (NC) groups, patients with SZ have disruption of small-world networks during a resting-state based on EEG [28,29,30] and fMRI results [6,31]. Disruptions in the small world network were found in several cortical regions, including the prefrontal, parietal, and temporal lobes [6,28,30,31]. Several fMRI studies have suggested significant correlations between psychotic symptoms and decreased efficiency of brain networks. The negative symptom score on the PANSS has a negative correlation with global efficiency and a positive correlation with mean path length [32]. A decrease in connectivity between the cerebellum and dorsolateral prefrontal cortex was found to correlate with increased negative symptoms [33]. Positive symptoms have shown a positive correlation with functional connectivity in the posterior cingulate and middle temporal regions [34]. At the same time, disorganization symptoms may be correlated with impaired functioning of frontoparietal networks [35]. However, these correlation tendencies between the topological indicators of brain networks and the clinical severity of SZ have not replicated in other studies [29,31,36].

In this study, we aimed to diagnose SZ compared to NC by brain network connectivity features of EEG during a resting-state. In addition, we tried to classify patients with SZ into high vs. low symptom groups for positive, negative, and cognitive/disorganization symptoms.

## 2. Materials and Methods

### 2.1. Participants

A total of 119 SZ patients (male: 53, female: 66, age: 36.26 ± 12.40 (range: 16–61)) were recruited for this study; they underwent the Structured Clinical Interview for DSM-V Axis I disorders (SCID-I) [37]. The PANSS was used to evaluate patients’ psychiatric symptoms [12]. Patients with a lifetime history of central nervous system disease, alcohol or drug abuse, mental retardation, or head injury with loss of consciousness, and patients with current Axis II disorders were excluded from the study. Among the 119 SZ patients, 25 were drug naive and 94 were taking antipsychotic medications (aripiprazole: *n* = 11, amisulpride: *n* = 10, blonanserin: *n* = 6, clozapine: *n* = 5, haloperidol: *n* = 1, olanzapine: *n* = 16, paliperidone: *n* = 11, quetiapine: *n* = 10, risperidone: *n* = 22, ziprasidone: *n* = 1, zotepine: *n* = 1) during the course of the study.

A total of 119 NC participants (male: 51, female: 68, age: 36.67 ± 11.66 (range: 20–61)) were recruited from local community advertising. They underwent an initial screening interview and were excluded if they had shown any identifiable neurological disorder, head injury, or any personal or family history of psychiatric illness. The further exclusion was processed through the Structured Clinical Interview for DSM V for Axis I Psychiatric Disorders [37]. All procedures followed were approved by the Institutional Review Board (IRB) at Inje University Ilsan Paik Hospital, Republic of Korea (2018-12-012-002), and were executed following the guidelines and regulations of the board. All participants provided written informed consent, and legal guardians provided informed consent if the participants were under the age of 18. Demographic data of the SZ and NC groups and the mean and standard deviation (SD) of psychiatric symptom severity scores in the SZ group are reported in Table 1.

### 2.2. SZ Subtype Classification according to Symptom Severities

The PANSS [12,38] assesses the severity of the two common types of symptoms (positive and negative) of SZ and the general psychopathology of the patient based on the interview and reports of family members. To assess the cognitive/disorganization symptoms, a five-factor model of the PANSS was used based on evidence from the factor analysis studies [20,21]. Each symptom was measured by several items, positive (P1, P3, P5, P6), negative (N1, N2, N3, N4, N6, G7, G16), cognition/disorganization (P2, N5, G9, G10, G11, G13, G15), excitement (P4, P7, G8, G12, G14), and depression/anxiety (G2, G3, G4, G6) [21].

The general PANSS positive score of the SZ group was 19.21 ± 12.40 (a negative score was 19.93 ± 6.66), and the general score was 41.75 ± 13.80. The five-factor model of the PANSS positive score was 11.56 ± 5.26, and a negative score was 19.60 ± 6.95. The cognitive/disorganization score was 17.69 ± 7.16, excitement score was 12.72 ± 5.86, and depression/anxiety score was 11.79 ± 3.84.

**Figure 1:**
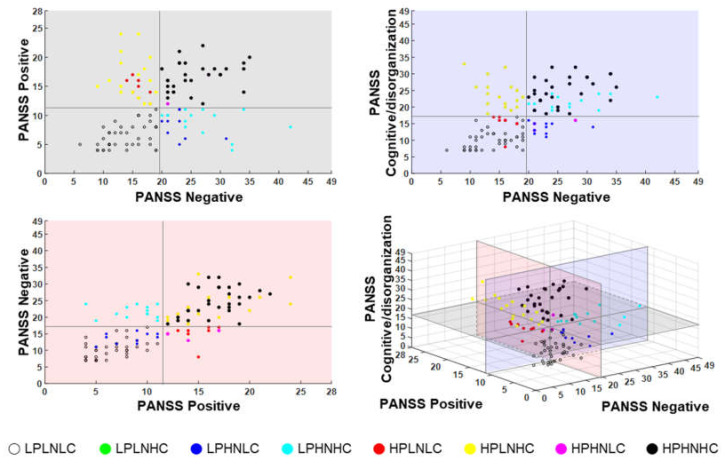
The positive and negative syndrome scale (PANSS) subscale scores of all schizophrenia (SZ) patients. LP: low positive; LN: low negative; LC: low cognitive/disorganization; HP: high positive; HN: high negative; HC: high cognitive/disorganization.

SZ patients were divided into separate groups based on the median score of the PANSS positive, negative, and cognitive/disorganization scores. The high PANSS positive (HPSZ, *n* = 57) group and the low PANSS positive (LPSZ, *n* = 62) group were divided based on the median score 11 of the PANSS positive subscale. The high PANSS negative (HNSZ, *n* = 55) group and the low PANSS negative (LNSZ, *n* = 64) group were divided based on the median score of 19 of the PANSS negative subscale. The high PANSS cognitive/disorganization (HCSZ, *n* = 59) group and the low PANSS cognitive/disorganization (LCSZ, *n* = 60) group were divided based on the median score of 17 of the PANSS cognitive/disorganization subscale. Figure 1 presents the distribution of SZ patients according to the PANSS subscale scores. Three pairs of subgroups did not show any significant differences between gender and education. Gender: HPSZ (male: 30, female: 27) vs. LPSZ (male: 23, female: 39), *p* = 0.064; HNSZ (male: 25, female: 30) vs. LNSZ (male: 28, female: 36), *p* = 0.499; HCSZ (male: 31, female: 28) vs. LCSZ (male: 22, female: 38), *p* = 0.059. Education: HPSZ: 12.93 ± 2.82 vs. LPSZ: 13.16 ± 2.98, *p* = 0.536; HNSZ: 12.81 ± 2.77 vs. LNSZ: 13.25 ± 3.00, *p* = 0.582; HCSZ: 12.73 ± 2.88 vs. LCSZ: 13.35 ± 2.89, *p* = 0.898.

### 2.3. EEG Data Acquisition and Analysis

Participants sat on a chair in a room where the ambient noise was blocked. The resting-state quantitative EEG was recorded with participants’ eyes closed for four minutes. EEG signals were recorded using a Quick Cap with 62 Ag-AgCl electrodes, which were placed according to the extended 10–20 system, and NeuroScan SynAmps (Compumedics USA, El Paso, TX, USA). The vertical electrooculogram (EOG) was recorded with the electrodes attached above and below the left eye, while the horizontal EOG was recorded with the electrodes attached to the outer canthus of each eye. We recorded EEG data with a 0.1–100 Hz band-pass filter at a sampling rate of 1000 Hz and removed 60 Hz noise using a notch filter. We analyzed the resting EEG data during the eyes closed session using CURRY 7 (Compumedics USA, Charlotte, NC, USA), a commonly used neuroimaging and analysis tool for EEG pre-processing. A trained inspector identified and manually removed the gross artifacts. The removal of the artifacts caused by eye movement and blinks was conducted using the covariance analysis of CURRY 7 [39]. The pre-processed EEG data were divided into two-second long epochs. Any epochs containing artifacts with the amplitude exceeding ±100 μV at any site, overall 62 electrodes, or a theta power/alpha power ratio > 1 were excluded from the analysis. In the power spectral analysis, we employed the periodogram function in MATLAB R2017b (MathWorks, Natick, MA, USA) to estimate the power spectral density of each epoch. After artifact rejection, 30 epochs were randomly selected for the following analysis.

### 2.4. Feature Extraction

In the study, the source-level cortical functional connectivity network was obtained. We estimated the time series of source activity using minimum norm estimation (MNE), and the synchronization between each pair of cortical sources measuring the phase-locking value (PLV). Values of the clustering coefficient (CC) and path length were evaluated for individual cortical functional networks during the resting-state with eyes closed.

Source localization was performed using Brainstorm [40], an open-source brain imaging tool (http://neuroimage.usc.edu/brainstorm). A three-layer boundary element method (BEM) model expressed in anatomical MNI template Colin 27 was used to compute the lead field matrix. We obtained cortical current density values at 15,002 cortical vertices for all time points of each epoch and extracted 148 dipole sources as evenly as possible from the original cortical surface model based on the Destrieux atlas [41]. The 148 sub-regions of the Destrieux atlas could be categorized into seven regions: the frontal lobe, insula, temporal lobe, occipital lobe, tempo-occipital lobe, parietal lobe, and the limbic lobe. The time series data at each of the 148 cortical locations were band-pass filtered and grouped into eleven frequency bands (delta (1–4 Hz), theta (4–8 Hz), alpha (8–12 Hz), alpha1 (8–10 Hz), alpha2 (10–12 Hz), beta (12–30 Hz), beta1 (12–18 Hz), beta2 (18–22 Hz), beta3 (22–30 Hz), beta4 (18–30 Hz) and gamma (30–55 Hz)) [42,43].

A network is essentially several nodes connected at their edges. The nodes are the brain sub-regions, and the PLV quantifies the edges among the potential pairs of cortical regions of interest [44]. The PLV measures phase synchronization between two different electrode locations but were recorded during the same time interval and the same frequency band [44]. Even when their amplitudes are not correlated, these phases can be synchronized [45]. Stationarity-independent, the PLV focuses purely on phase and ranges from 0 to 1. Values close to 1 mean the two signals are synchronized and show a constant time lag. Signals with values close to zero are temporally independent. The PLV was chosen as the measure of synchronization, since it ranges from 0 to 1 and consequently requires no additional modifications to reflect connection strength in weighted network analysis.

In this study, we applied graph theory to perform weighted network analysis. As aforementioned, a network is composed of several nodes, which are connected by edges. The CC indicates the degree to which a node is clustered with its neighboring nodes. The CC was calculated for the entire network. The path length indicates the overall connectedness of the whole network, and is calculated as the sum of the lengths between two nodes in the entire network. The weighted CC indicates the functional segregation of a network, while the path length refers to the functional integration [46]. For the respective nodes, the CC was first calculated (described as local level results), and then an average was created for all of the cortical regions concerned (i.e., global level). Given that it is defined purely at the global level, no values of path length at the local level were available [47].

### 2.5. Feature Selection and Classification

**Figure 2:**
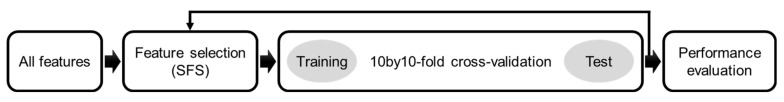
The flow process of the overall analysis procedures. SFS: sequential forward selection.

The objective of the study was to distinguish not only between the SZ group and NC, but also among the subtypes of SZ: HPSZ, LPSZ, HNSZ, LNSZ, HCSZ, and LCSZ. Hence, we set four different classification pairs: (1) SZ-NC; (2) HPSZ-LPSZ; (3) HNSZ-LNSZ; and (4) HCSZ-LCSZ. In discrimination analysis, a source-level feature set (1650 features) was tested. To select features for classification, a wrapped feature selection technique named sequential forward selection (SFS) was applied. SFS is a bottom-up searching technique. It first selects the best feature according to a cost function. When it is combined with every remaining feature, it selects the best pair with the greatest value evaluation as the new starting set. Subsequently, this chosen pair is combined with each of the remaining variables, forming triads. Then, the triad that offers a greater value in the evaluation criteria is selected. The process continues until it meets the criterion. The search stops when a set of variables does not improve the results of the cost function. The number of selected features ranged from 1 to 30. The classification accuracy was evaluated using 10 by 10-fold cross-validation, which repeats a 10-fold cross validation 10 times to obtain more generalized classification accuracies, with the linear discriminant analysis (LDA) classifier [48,49], for each feature set. In addition, we computed the statistically significant threshold of classification accuracies by using the MATLAB (Mathworks Inc., Natick, MA, USA) function *binoinv*: $\mathrm{St}\left(\alpha \right)=binoinv\left(1-\alpha ,n,1/c\right)\times 100/n$ (*n*: sample size, *c*: the number of classes, *α*: significance level) [50]. Figure 2 illustrates the overall analysis procedures in this study.

## 3. Results

**Figure 3:**
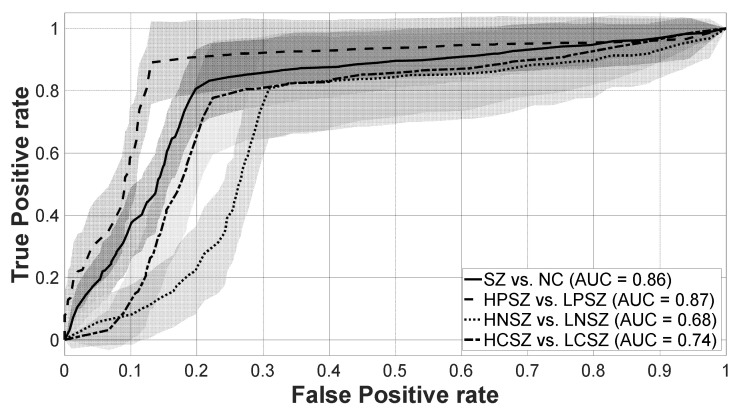
Receiver operating characteristic (ROC) curves in each pair of classifications. Solid, dashed, dotted, and dash-dotted lines respectively mean ROC curves at SZ vs. NC, HPSZ vs. LPSZ, HNSZ vs. LNSZ, and HCSZ vs. LCSZ. Lines represent the average of the ROC curves in 10 by 10-fold cross-validation, and areas represent standard deviation values. SZ: schizophrenia, NC: normal control, HPSZ: high positive SZ, LPSZ: low positive SZ, HNSZ: high negative SZ, LNSE: low negative SZ, HCSZ: high cognitive/disorganization SZ, LCSZ: low cognitive/disorganization SZ, AUC: area under the curve.

The highest classification accuracy for each classification pair was as follows: (1) SZ vs. NC: 80.66%; (2) HPSZ vs. LPSZ: 88.10%; (3) HNSZ vs. LNSZ: 75.25%; and (4) HCSZ vs. LCSZ: 77.78%. As the theoretical chance level (100/2 = 50%) is defined for an infinite number of data, we used the binomial cumulative distribution [50] to calculate statistical significance thresholds for decoding accuracy, the results of which were 55.46% (*n* = 238, two classes and *p* < 0.05) and 57.14% (*n* = 119, two classes and *p* < 0.05). Table 2 summarized the best mean classification accuracy, specificity, and sensitivity in each pair of classifications, and receiver operating characteristic (ROC) curves are shown in Figure 3.

Among the seven brain regions, features of the frontal and parietal lobes were frequently selected with the best classification accuracies. When classifying the SZ vs. NC, the most frequently selected were features of the frontal lobe, followed by the features of the occipital > limbic > temporal = parietal lobe. When classifying the HPSZ vs. LPSZ, the features of the frontal lobe were the most frequently selected, followed by the features of the tempo-occipital > temporal = occipital = parietal lobe. When classifying the HNSZ vs. LNSZ, the features of the frontal, tempo-occipital, and parietal lobes were the most frequently selected, followed by the features of the insula. When classifying the HCSZ vs. LCSZ, the features of the parietal lobe were the most selected, followed by the features of the frontal lobe > temporal lobe = limbic. The selected features of the brain regions in each classification pair are summarized in Table 2.

Among 11 frequency bands, when classifying the SZ vs. NC, the features of the theta and beta3 bands were the most frequently selected, followed by the features of the delta > alpha > beta2 band. When classifying the HPSZ vs. LPSZ, the features of the alpha band were the most frequently selected, followed by the features of the delta > theta = alpha1 = beta4 = gamma. When classifying the HNSZ vs. LNSZ, the features of the alpha2 band were the most frequently selected, followed by the features of the delta = theta = beta1 = beta4 = gamma band. When classifying the HCSZ vs. LCSZ, the features of the beta2 band were the most frequently selected, followed by the features of the delta = alpha = beta > gamma band. Table 2 shows a summary of the selected features of frequency bands in each classification pair.

Interestingly, the least number of features were selected to classify HNSZ vs. LNSZ groups. The best classification accuracy was 75.25% when using the seven features (CC of the right supramarginal gyrus, left anterior transverse collateral sulcus, right precuneus, left inferior segment of the circular sulcus of the insula, left posterior transverse collateral sulcus, left triangular part of the inferior frontal gyrus, and the marginal branch of the cingulate sulcus). The best classification accuracy, specificity, sensitivity, and selected features in each step of the classification of the HNSZ vs. LNSZ groups are summarized in Table 3.

## 4. Discussion

In this study, we aimed to diagnose SZ compared to NC and classify the subtypes of SZ according to symptom severity. Source-level brain network analysis of resting-state EEG was used as the feature in the machine learning classification. Classification of SZ and NC and symptom-based SZ subgroups of SZ classification showed sufficiently high classification accuracies. Features from the frontoparietal regions were frequently selected for the best classification of SZ patients compared to NC.

Classification of SZ and NC and symptom-based SZ subgroups of SZ classification were completed with sufficiently high classification accuracies. A growing number of studies has sought to differentiate SZ patients and NC by using machine learning approaches with brain signal biomarkers during a resting-state. Some fMRI and EEG studies used functional alterations in resting-state brain signals as features for classification [51,52,53,54]. The classification accuracies of SZ were about 92.86% (*n* = 28) with resting-state fMRI [53], 91.0% (*n* = 18) with fMRI [52], 97.1% (*n* = 26) with fMRI [51], and 92.0% (*n* = 45) with EEG [54]. However, larger sample-based diagnostic models tend to demonstrate classification accuracies that fall below 80% [55,56,57,58]. Many have observed the phenomenon that “smaller-*n* studies reach higher prediction accuracy of SZ with neuroimaging data” [59]. The higher cross-validated accuracy obtained from smaller samples may fail to detect the existing heterogeneity of the disorder.

Among the symptomatic classifications, positive symptoms showed the highest classification accuracy (88.10%). The brain network connectivity features in the frontal lobe were selected as the best features. It has already been well-known that positive symptoms are the major symptoms of SZ and are easier to recognize than other symptoms [60]. SZ presents a range of functional changes in the frontal lobe. Within the SZ group, the gray matter volume in the bilateral frontal lobe shows a negative correlation with hallucination [61]. SZ groups also tend to show a deficient dopamine release capacity in the dorsolateral prefrontal cortex [62]. Therefore, obvious pathological symptoms and their robust underlying brain network abnormality may contribute to the highest classification accuracy of positive symptoms compared to other symptoms of SZ.

Negative symptom severity showed the lowest classification accuracy (75.25%). However, the number of selected features was the lowest at seven when the highest classification accuracy was achieved. The selected features were mainly located in the frontal (triangular part of the inferior frontal gyrus and marginal branch of the cingulate sulcus), tempo-occipital (the anterior transverse collateral sulcus and posterior transverse collateral sulcus), parietal (the supramarginal gyrus and precuneus), and insula (the inferior segment of the circular sulcus of the insula) regions. Negative symptoms are usually considered as stable traits in the pathology of SZ [63,64,65,66,67,68,69], and respond poorly to medication [70]. It may be difficult for novice physicians with a limited number of sessions with the patient to recognize negative symptoms. The smallest number of features (*n* = 7) were used to classify negative symptoms (high vs. low). This supports that negative symptoms could be a trait and core pathology of SZ.

Features in the frontoparietal regions were frequently selected for the best classification of patients with SZ compared to NC. Altered functional neural circuits, rather than the dysfunction of a single brain structure, are involved in SZ [71]. The frontal and parietal regions are known as important pathological regions related to SZ [72,73,74,75]. The frontal lobe is critical for social-emotional and insight processing. Furthermore, there is greater hypofrontality in SZ than in NC [76], and changes in oxygenated hemoglobin in the frontal cortex are positively correlated to the severity of psychotic symptoms in SZ patients [76,77,78,79]. The parietal lobe is associated with a wide range of cognitive functions [80].

Our study has some limitations that need to be addressed. Most of the SZ patients involved were taking medications. We could not control for the possible effects of all psychotropic medications. Participants in our study were mostly chronic patients. First onset schizophrenia patients may show characteristics of brain EEG networks that differ from chronic SZ patients. Lastly, there is a lack of specific negative symptomatology analysis, and we did not use a specific neurocognitive screening tool for cognitive measurement when creating SZ subgroups.

Our research is the first attempt to diagnose SZ compared to NC and classify the subtypes of SZ according to symptom severity. We achieved acceptable classification accuracies by simply using resting-state EEG. Our method could be a promising approach in the computer-assisted diagnosis of SZ.

## Figures and Tables

**Table 1:** Demographic data of the schizophrenia (SZ) and normal control (NC) groups and the mean and standard deviation (SD) of psychiatric symptom severity scores in the SZ group.

	SZ (*n* = 119)	NC (*n* = 119)	*p*-Value
	Mean ± SD or *n*		
Age (years)	36.26 ± 12.40	36.67 ± 11.66	0.792
Sex			0.794
Male	53	51	
Female	66	68	
Education (years)	13.05 ± 2.89	13.55 ± 2.89	0.186
Number of hospitalization	2.43 ± 2.88		
Duration of illness (years)	9.93 ± 9.21		
Dosage of antipsychotics (chlorpromazine equivalent, mg)	887.83 ± 1110.95		
Positive and negative syndrome scale (PANSS)			
Positive	19.21 ± 8.69		
Negative	19.93 ± 6.66		
General	41.75 ± 13.80		
Total	80.89 ± 25.31		
Five-factor model of the PANSS			
Positive	11.56 ± 5.26		
Negative	19.60 ± 6.95		
Cognitive/disorganization	17.69 ± 7.16		
Excitement	12.72 ± 5.86		
Depression/anxiety	11.79 ± 3.84		

**Table 2:** The best mean classification accuracy, specificity, and sensitivity in each classification pair. The brain region and frequency band ranking of selected features in each classification pair. SZ: schizophrenia, NC: normal control, HPSZ: high positive SZ, LPSZ: low positive SZ, HNSZ: high negative SZ, LNSZ: low negative SZ, HCSZ: high cognitive/disorganization SZ, LCSZ: low cognitive/disorganization SZ.

Two-Classes Classification	Accuracy (%)		Sensitivity (%)			Specificity (%)			# of Features		
SZ (*n* = 119) vs. NC (*n* = 119)	80.66		78.83			82.48			27		
HPSZ (*n* = 57) vs. LPSZ (*n* = 62)	88.10		88.40			87.77			19		
HNSZ (*n* = 55) vs. LNSZ (*n* = 64)	75.25		80.76			68.50			7		
HCSZ (*n* = 59) vs. LCSZ (*n* = 60)	77.78		77.83			77.80			27		
**Selected features ranking** **(brain region)**	**1st**		**2nd**			**3rd**			**4th**		**5th**
SZ vs. NC	Frontal	>	Occipital		>	Limbic		>	Temporal	=	Parietal
HPSZ vs. LPSZ	Frontal	>	Tempo-Occipital		>	Temporal		=	Occipital	=	Parietal
HNSZ vs. LNSZ	Frontal	=	Tempo-Occipital		=	Parietal		>	Insula		
HCSZ vs. LCSZ	Parietal	>	Frontal		>	Temporal		=	Limbic		
**Selected features ranking** **(frequency band)**	**1st**		**2nd**		**3rd**		**4th**		**5th**		**6th**
SZ vs. NC	Theta	=	Beta3	>	Delta	>	Alpha	>	Beta2		
HPSZ vs. LPSZ	Alpha	>	Delta	>	Theta	=	Alpha1	=	Beta4	=	gamma
HNSZ vs. LNSZ	Alpha2	>	Delta	=	Theta	=	Beta1	=	Beta4	=	gamma
HCSZ vs. LCSZ	Beta2	>	Delta	=	Alpha	=	Beta	>	Gamma		

**Table 3:** The best classification accuracy, specificity, sensitivity, and selected features in each step of the classification of the negative symptom (HNSZ vs. LNSZ). HNSZ: high negative schizophrenia (SZ), LNSZ: low negative SZ, R: right, L: left.

#	Accuracy (%)	Sensitivity (%)	Specificity (%)	Frequency Band	Brain Region
1	63.69	70.52	56.03	Delta	Supramarginal gyrus R
2	69.83	75.98	62.60	Alpha2	Anterior transverse collateral sulcus L
3	71.99	76.38	66.90	Gamma	Precuneus (medial part of P1) R
4	73.90	80.05	67.10	Beta1	Inferior segment of the circular sulcus of the insula L
5	74.67	80.81	67.33	Theta	Posterior transverse collateral sulcus L
6	75.13	80.33	68.93	Alpha2	Triangular part of the inferior frontal gyrus L
7	75.25	80.76	68.50	Beta4	Marginal branch (or part) of the cingulate sulcus R
